# A novel effect of Noscapine on patients with massive ischemic stroke: A pseudo-randomized clinical trial

**Published:** 2015-01-05

**Authors:** Massoud Mahmoudian, Mohammad Rezvani, Mohammad Rohani, Foozya Benaissa, Mehdi Jalili, Shadi Ghourchian

**Affiliations:** 1 Department of Pharmacology, School of Medicine, Tehran University of Medical Sciences, Tehran, Iran; 2 Department of Neurology, School of Medicine, Tehran University of Medical Sciences, Tehran, Iran; 3 Department of Neurology, School of Medicine, Iran University of Medical Sciences, Tehran, Iran

**Keywords:** Noscapine, Massive Ischemic Stroke, Treatment, Clinical Trial

## Abstract

**Background: **Massive ischemic stroke causes significant mortality and morbidity in stroke patients. The main treatments for massive ischemic stroke are recombinant tissue plasminogen activator (rtPA), craniotomy, and endovascular interventions. Due to destructive effects of bradykinin on the nervous system in ischemic stroke, it seems reasonable that using Noscapine as a Bradykinin antagonist may improve patients’ outcome after ischemic stroke. The effect of Noscapine on massive ischemic stroke was shown by the previous pilot study by our group. This pseudo-randomized clinical trial study was designed to assess the result of the pilot study.

**Methods:** Patients who had clinical symptoms or computed tomography scan indicative of massive stroke (in full middle cerebral artery territory) were entered to the study. The cases received the drugs according to their turns in emergency ward (pseudo-randomized). The patient group received Noscapine, and the control group received common supportive treatments. The patients and data analyzer were blinded about the data. At the end of the study, to adjust confounding variables we used logistic regression.

**Results: **After 1-month follow-up, 16 patients in the control group and 11 patients in the case group expired (P = 0.193). Analyzing the data extracted from Rankin scale and Barthel index check lists, revealed no significant differences in the two groups.

**Conclusion: **Despite the absence of significant statistical results in our study, the reduction rate of 16% for mortality rate in Noscapine recipients is clinically remarkable and motivates future studies with larger sample sizes.

## Introduction

Although the stroke is the third cause of death in the world but its treatment is limited to recombinant tissue plasminogen activator (rtPA) and mechanical methods (arterial recanalization). Using these methods needs special settings, and the time of their administration is limited. Furthermore, some countries do not afford these treatments.^1^^-^^3^

The neuroprotective treatments have been proved to be effective in animal models, but the trials have not proved these effects in the human being.^4^^-^^8^

Noscapine is an alkaloid microtubule binding agent initially derived from the opium plant, but it does not have sedative, euphoric, palliative, and respiratory depressant effects.^9^ This novel substance has been used as a cough suppressant.^10^ Noscapine is taken orally and can cross blood brain barrier easily.^7^ The antitussive activity is attributed to central mediated mechanisms.^11^

Bradykinin is an autacoid substance that exists physiologically in the body.^12^ This substance induces arterial dilation through direct effect on muscular layer and indirectly by releasing the endothelial derived releasing factors such as nitric oxide and prostaglandin I_2_. It has an adverse effect on veins and causes venous constriction. It also stimulates edema and inflammation.^12^ In a previous study, it was shown that the Noscapine inhibits the contractive effect of bradykinin in pig’s intestine and rat’s vasodefran.^13^ It was hypothesized that the antitussive effect of the drug is due to suppression of bradykinin (FR190997).^14^

Microtubule binding agents also have anti-angiogenic activity by disrupting the endothelial tubule formations. Noscapine inhibits vascular endothelial growth factor (VEGF) and suppress neovascularization. The mechanism of reducing the neovascularization was suggested by Newcomb et al.^15^

Due to the inflammatory and destructive effects of bradykinin in ischemic stroke, it seems reasonable that using Noscapine as a Bradykinin antagonist may improve patients’ outcome after ischemic stroke. The drug’s effect on reducing mortality rate of mice with cerebral injuries secondary to hypoxic-ischemic attack, and also on reducing the mortality rate of patients with large acute ischemic stroke in a pilot study,^16^ asserts designing the new study with larger sample size to understand whether Noscapine is effective on mortality rate of patients with acute massive ischemic stroke.

## Materials and Methods

This study was conducted in the review board of the Tehran University of Medical Sciences, Iran, and the ethical approval was confirmed by Iranian Registry of Clinical Trials. This is a Primary Registry in the World Health Organization (WHO) Registry Network with the help from the Ministry of Health and Medical Education. The study was performed at Rasoul-e-Akram Hospital, one of the main referral neurology centers of Tehran. All researchers respected to patients’ data and the declaration of Helsinki. The authors did not have any conflict of interests in this study.

This study was a pseudo-randomized clinical trial that was conducted paralleled.

The patients with a diagnosis of large ischemic stroke both clinically and by neuroimaging [brain computed tomography (CT) scan] were entered to the study .We defined large ischemic strokes as strokes with involvement of total middle cerebral artery territory. The patients with normal initial CT scans underwent the second CT scan 24 h after the first one. If the diagnosis was not confirmed, the patient was excluded from the study.

Patients with modified Rankin scale > 2 before the presentation of ischemic stroke were excluded from the study. Other expressed inclusion and exclusion criteria were considered prior to the initiation.

Inclusion criteria were defined as the age of 18 years old and older; the interval of 12 h or less from symptom onset to admission and the level of consciousness > 2; limb movement > 7 and the total score > 7 by assessing the National Institutes of Health Stroke Scale (NIHSS).^17^

Since at the time of the study rtPA was not used in our center for stroke patients, none of the patients in case or control group received rtPA.

Exclusion criteria were defined as modified Rankin scale more than 2 before the presentation of stroke, relief of neurologic defects within the first minutes, ,suspicious of septic meningitis or subarachnoid hemorrhage or neurologic defects caused by seizures and could not be differentiated from Todd’s phenomenon. Also patients with probable or confirmed brain masses, congestive heart failure, renal or liver diseases, proliferative retinopathy, cancer, head trauma, mandatory mechanical ventilation, unstable vital signs and psychological disorders which interfered with mental state evaluation were all excluded from the study.

In this study, allocation was based on a pseudo randomization process in which patients with odd numbers considered as the control group and the others with even numbers received the novel drug added to the routine regime (based on their consecutive assigned number in emergency ward). The patient group received Noscapine and common supportive treatments, and the control group received only common supportive treatments. The data analyzer was completely blinded to the treatment strategies. Noscapine was used as syrup in conscious patients and via nasogastric tube in unconscious patients. The syrup was made by Capval-saft Company, Germany. The applied dosage was considered 50 mg/three times a day for 5 days. The dosage was assimilated to the previous dosage used in patients with coughing.

To evaluate the patients we used:

1. Brain CT scan for confirmation of massive ischemic stroke
2. Physical examination for the diagnosis of ischemic stroke and filling out the NIHSS at the emergency ward, 1 week and 1 month after receiving the drugs.
3. The checklists of Rankin scale and Barthel index for evaluating functional disabilities 1 week after receiving the drugs.^18^

Though the main aim of this study was comparing the mortality rate in patients with massive ischemic stroke who received Noscapine with those who received usual drugs, the mortality rate was estimated after 1 month.

Sampling was performed conveniently in the patients with the diagnosis of massive brain ischemic stroke. According to the previous studies, α and β error were considered 0.05 and 0.1 respectively. By using sampling tests, the number of cases was estimated 16 in each group, but for increasing the power of the study the sample size was considered 30 in each group.

The data were entered to the SPSS software (version 18, SPSS Inc., Chicago, IL, USA). For comparing the qualitative variables such as mortality rate in the two groups, we used chi-square test. For comparing the disability scores, Rankin scale, and Barthel index, if the data were distributed parametrically, we used student's T-test or its non-parametric equivalent (Mann–Whitney U-test). Logistic regression was applied on parameters with significant or near significant differences for further adjustment.

## Results

The study included 30 patients in each group and none of them were excluded during the study; whereas, 13 of them in the case group and 14 of them in the control group were men. Case group was defined as the patients who received Noscapine. The mean age in the case and control group was 69.5 ± 4.34 and 71.1 ± 4.97 years old, respectively.

Among Noscapine recipients, the left brain hemisphere was involved in 16 patients and right hemisphere in 14 patients. In the control group, the left hemisphere was involved in 13 patients and right hemisphere in 17 patients. The prevalence of demographic characteristics and some initial risk factors in each group is illustrated in table 1. The analysis of these risk factors revealed no significant differences between the two groups.

After 1 month follow-up, 16 patients (53%) in the control group and 11 patients (37%) in the case group expired. The chi-square test revealed no significant difference between the two groups (P = 0.193).

Further adjustment analysis by logistic regression showed no significant different in mortality rate between the two groups (Table 2).

Of all 60 patients, pneumonia was seen in 11 cases, ischemic heart attack in 8, cardiac arrhythmia in 3, deep vein thrombosis in 2, brain herniation in 1, gastrointestinal bleeding in 1, and diabetic ketoacidosis in 1 case were the main causes of patients’ death at the end of the study.

Analyzing the data extracted from Rankin scale and Barthel index check lists, revealed no significant differences between the two groups (Table 3). 

## Discussion

Although the previous pilot study in patients with massive ischemic stroke revealed significant decrease in mortality rate in Noscapine recipients,^16^ our study with larger sample size did not show any significant difference between the two groups. Further, analyses from the Rankin scale and Barthel index check lists showed the drug could not reduce the morbidity in these patients.

In studies performed in 1992 and 2004, it was shown that radiolabeled Noscapine crosses the blood-brain barrier and binds to the neurons of the central nervous system.^19^

Hitherto, the anticancer/anti-proliferative effect of Noscapine has been discussed. The drug increases microtubules pause phase and also induces apoptosis through mitochondrial pathways.^8^^,^^9^^,^^20^^-^^22^ Suppressing microtubule dynamics without the disturbing microtubule polymer figure reduces toxic effects on normal tissues while the anticancer activity retains.^10^^,^^22^ Although specific side-effect has not been reported noticeably, chest pain was reported in one article.^23^

**Table 1:** Demographic characteristics

**Characteristic**	**Control group (%)**	**Noscapine group (%)**	**P**
Age			
Range	48-85	38-90	0.347
Mean	71.0	69.5	0.521
Female	53.0	43.5	0.347
Male	47.0	56.5	0.212
HTN	53.4	63.4	0.432
Smokers	23.3	13.3	0.314
Previous stroke	26.7	20.0	0.541
HLP	6.7	3.3	0.550
Diabetics	36.7	26.7	0.404
IHD	46.6	30.0	0.183
AF	26.7	20.0	0.541
MS	3.3	13.3	0.814

HTN: Hypertension; HLP: Hyperlipidemia; IHD: Ischemic heart disease; AF: Atrial fibrillation; MS: Mitral stenosis

**Table 2:** The relationship between mortality rate and different risk factors

**Factors**	**Dead**	**Alive**	**P**
Sex			
Male	15	12	0.938
Female	18	15	
HTN			
+	15	10	0.511
-	18	17	
DM			
+	21	20	0.387
-	12	7	
IHD			
+	21	16	0.729
-	12	11	
CVA			
+	24	22	0.425
-	9	5	
HLP			
+	31	26	0.687
-	2	1	
AF			
+	26	20	0.668
-	7	7	
Smoking			
+	25	24	0.191
-	8	3	
MS			
+	30	24	0.814
-	3	2	

HTN: Hypertension; DM: Diabetics mellitus; IHD: Ischemic heart disease; CVA: Cerebrovascular accidents; HLP: Hyperlipidemia; AF: Atrial fibrillation; MS: Mitral stenosis

**Table 3:** Functional (Barthel index) and disability (Modified Ranki scale) outcomes after 1 month

**Functional and disability outcomes**	**Control group**	**Noscapine group**
a. Barthel index (%)		
95-100	0	0
55-90	0	1 (3.4)
0-50	16 (53.4)	18 (60.0)
Died	14 (46.6)	11 (36.6)
b. Modified Rankin scale (%)		
0-1	0	0
2-3	0	4 (13.4)
4-5	16 (53.4)	15 (50.0)
Died	14 (46.6)	11 (36.6)

An in vitro study in 2008 planned for assessing the neuro-protective potential of Noscapine that demyelinating effect of vincristine used in patients with acute lymphoblastic leukemia was decreased when Noscapine was added to the regime.^7^

Electrically induced coughs that mimic the medicine induced coughs are relatively suppressed by Noscapine, by means of affecting autonomic nervous system.^11^

Newcomb et al. showed that Noscapine disrupts the functional pathway of hypoxia-inducible factor-1 (HIF-1) in tumors especially glioma. They also demonstrated that these effects are accompanied by inhibition of HIF-1α expression that leads to decreased secretion of VEGF.^15^

Landen et al., in 2004, showed that Noscapine hinders the growth of a highly aggressive mouse glioma. They revealed that Noscapine may be a novel hope for treating aggressive glioma that does not respond to chemotherapy.^19^

In another study in Iran, in 2003, it was shown that mice’s brain edema was significantly reduced by Noscapine.^14^

In our study, although the risk factors (diabetes mellitus, ischemic heart disease, previous ischemic stroke, hyperlipidemia, atrial fibrillation, and smoking) were not completely matched between the two groups but using logistic regression for adjustment, there revealed no significant difference between mortality rates between groups.

Although the location of the study, the company of the medicine, and the inclusion and exclusion criteria in this study were similar to the previous pilot study, the result was different. Unlike the previous pilot study, we did not find any significant correlation between atherosclerosis risk factors, the involved hemisphere and the demographic factors such as age and sex with the mortality rate in the groups. According to the differences between the studies, future evaluations with more sample size are recommended.

Despite the absence of significant decrease in mortality rate in the Noscapine group, the reduction rate of 16% for mortality rate in Noscapine recipients (comparing to the control group) is clinically remarkable and motivates future studies with larger sample size for providing the higher power of study. Also, placebo usage in the control group is recommended.

## Conclusion

Massive ischemic stroke causes significant mortality and morbidity among stroke patients. Despite using rtPA, craniotomy and endovascular interventions for these patients, there is neither an effective neuroprotective nor an antiedema agents in these patients. Noscapine as an antibradykinin agent can be a potential candidate for decreasing brain edema, enhancing neuroprotection and thus reducing the mortality and morbidity of ischemic stroke. Although our study didn’t show a decrease in mortality rate among Noscapine recipients with massive ischemic stroke, further studies with larger sample volumes are needed to show the effects of this substance.

## Conflict of Interests

The authors declare no conflict of interest in this study.

## Notes

**How to cite this article: **Mahmoudian M, Rezvani M, Rohani M, Benaissa F, Jalili M, Ghourchian Sh. A novel effect of Noscapine on patients with massive ischemic stroke: A pseudo-randomized clinical trial. Iran J Neurol 2015; 14(1): 12-6.

## References

[1] Brott T, Bogousslavsky J (2000). Treatment of acute ischemic stroke. N Engl J Med.

[2] Hacke W, Kaste M, Bluhmki E, Brozman M, Davalos A, Guidetti D (2008). Thrombolysis with alteplase 3 to 4.5 hours after acute ischemic stroke. N Engl J Med.

[3] Jovin TG, Gupta R, Uchino K, Jungreis CA, Wechsler LR, Hammer MD (2005). Emergent stenting of extracranial internal carotid artery occlusion in acute stroke has a high revascularization rate. Stroke.

[4] Bath PM, Sprigg N (2007). Colony stimulating factors (including erythropoietin, granulocyte colony stimulating factor and analogues) for stroke. Cochrane Database Syst Rev.

[5] Gladstone DJ, Black SE, Hakim AM (2002). Toward wisdom from failure: lessons from neuroprotective stroke trials and new therapeutic directions. Stroke.

[6] Muir KW, Lees KR (2003). Excitatory amino acid antagonists for acute stroke. Cochrane Database Syst Rev.

[7] Hiser L, Herrington B, Lobert S (2008). Effect of noscapine and vincristine combination on demyelination and cell proliferation in vitro. Leuk Lymphoma.

[8] Savitz SI, Fisher M (2007). Future of neuroprotection for acute stroke: in the aftermath of the SAINT trials. Ann Neurol.

[9] Wade A (1977). Martindale, the Extra Pharmacopoeia.

[10] Ye K, Ke Y, Keshava N, Shanks J, Kapp JA, Tekmal RR (1998). Opium alkaloid noscapine is an antitumor agent that arrests metaphase and induces apoptosis in dividing cells. Proc Natl Acad Sci U S A.

[11] Balint G, Rabloczky G (1968). Antitussive effect of autonomic drugs. Acta Physiol Acad Sci Hung.

[12] Aramori I, Zenkoh J, Morikawa N, O'Donnell N, Asano M, Nakamura K (1997). Novel subtype-selective nonpeptide bradykinin receptor antagonists FR167344 and FR173657. Mol Pharmacol.

[13] Mahmoudian M, Mojaverian N (2001). Efffect of noscapine, the antitussive opioid alkaloid, on bradykinin-induced smooth muscle contraction in the isolated ileum of the guinea-pig. Acta Physiol Hung.

[14] Mahmoudian M, Siadatpour Z, Ziai SA, Mehrpour M, Benaissa F, Nobakht M (2003). Reduction of the prenatal hypoxic-ischemic brain edema with noscapine. Acta Physiol Hung.

[15] Newcomb EW, Lukyanov Y, Schnee T, Ali MA, Lan L, Zagzag D (2006). Noscapine inhibits hypoxia-mediated HIF-1alpha expression andangiogenesis in vitro: a novel function for an old drug. Int J Oncol.

[16] Mahmoudian M, Mehrpour M, Benaissa F, Siadatpour Z (2003). A preliminary report on the application of noscapine in the treatment of stroke. Eur J Clin Pharmacol.

[17] RANKIN J (1957). Cerebral vascular accidents in patients over the age of 60. II. Prognosis. Scott Med J.

[18] Muir KW, Weir CJ, Murray GD, Povey C, Lees KR (1996). Comparison of neurological scales and scoring systems for acute stroke prognosis. Stroke.

[19] Landen JW, Hau V, Wang M, Davis T, Ciliax B, Wainer BH (2004). Noscapine crosses the blood-brain barrier and inhibits glioblastoma growth. Clin Cancer Res.

[20] Ke Y, Ye K, Grossniklaus HE, Archer DR, Joshi HC, Kapp JA (2000). Noscapine inhibits tumor growth with little toxicity to normal tissues or inhibition of immune responses. Cancer Immunol Immunother.

[21] Landen JW, Lang R, McMahon SJ, Rusan NM, Yvon AM, Adams AW (2002). Noscapine alters microtubule dynamics in living cells and inhibits the progression of melanoma. Cancer Res.

[22] Zhou J, Panda D, Landen JW, Wilson L, Joshi HC (2002). Minor alteration of microtubule dynamics causes loss of tension across kinetochore pairs and activates the spindle checkpoint. J Biol Chem.

[23] Ottervanger JP, Wilson JH, Stricker BH (1997). Drug-induced chest pain and myocardial infarction. Reports to a national centre and review of the literature. Eur J Clin Pharmacol.

